# Detection of activated *KRAS* from cancer patient peripheral blood using a weighted enzymatic chip array

**DOI:** 10.1186/1479-5876-12-147

**Published:** 2014-05-26

**Authors:** Ming-Yii Huang, Hsueh-Chiao Liu, Li-Chen Yen, Jia-Yuan Chang, Jian-Jhang Huang, Jaw-Yuan Wang, Chao-Peng Hsiao, Shiu-Ru Lin

**Affiliations:** 1 Department of Radiation Oncology, Kaohsiung Medical University Hospital, Kaohsiung 807, Taiwan; 2 Department of Radiation Oncology, Faculty of Medicine, College of Medicine, Kaohsiung Medical University, Kaohsiung 807, Taiwan; 3 Cancer Center, Kaohsiung Medical University Hospital, Kaohsiung 807, Taiwan; 4 Personalized Medical Service Center, Division of Laboratory Medicine, Fooyin University Hospital, Pingtung 928, Taiwan; 5 Institute of Biomedical Sciences, National Sun Yat-San University, Kaohsiung 804, Taiwan; 6 Division of Medical Research, Fooyin University Hospital, Pingtung 928, Taiwan; 7 Division of Gastrointestinal and General Surgery, Department of Surgery, Kaohsiung Medical University Hospital, Kaohsiung 807, Taiwan; 8 Graduate Institute of Clinical Medicine, College of Medicine, Kaohsiung Medical University, Kaohsiung 807, Taiwan; 9 Department of Surgery, Faculty of Medicine, College of Medicine, Kaohsiung Medical University, Kaohsiung 807, Taiwan; 10 Department of Colorectal Surgery, Fooyin University Hospital, Pingtung 928, Taiwan

**Keywords:** Colorectal cancer, Lung cancer, Peripheral blood, Weighted enzymatic chip array (WEnCA), Activating *KRAS* Detection Chip

## Abstract

**Background:**

The *KRAS* oncogene was one of the earliest discoveries of genetic alterations in colorectal and lung cancers. Moreover, *KRAS* somatic mutations might be used for predicting the efficiency of anti-EGFR therapeutic drugs. The purpose of this research was to improve Activating *KRAS* Detection Chip by using a weighted enzymatic chip array (WEnCA) platform to detect activated *KRAS* mutations status in the peripheral blood of non-small-cell lung cancer (NSCLC) and colorectal cancer (CRC) patients in Taiwan.

**Methods:**

Our laboratory developed an Activating *KRAS* Detection Chip and a WEnCA technique that can detect activated *KRAS* mutation status by screening circulating cancer cells in the surrounding bloodstream. We collected 390 peripheral blood samples of NSCLC patients (n = 210) and CRC patients (n = 180) to evaluate clinical *KRAS* activation using this gene array diagnosis apparatus, an Activating *KRAS* Detection Chip and a WEnCA technique. Subsequently, we prospectively enrolled 88 stage III CRC patients who received adjuvant FOLFOX-4 chemotherapy with or without cetuximab. We compared the chip results of preoperative blood specimens and their relationship with disease control status in these patients.

**Results:**

After statistical analysis, the sensitivity of WEnCA was found to be 93%, and the specificity was found to be 94%. Relapse status and chip results among the stage III CRC patients receiving FOLFOX-4 plus cetuximab (n = 59) and those receiving FOLFOX-4 alone (n = 29) were compared. Among the 51 stage III CRC patients with chip negative results who were treated with FOLFOX-4 plus cetuximab chemotherapy, the relapse rate was 33.3%; otherwise, the relapse rate was 48.5% among the 23 out of 88 patients with chip negative results who received FOLFOX-4 alone. Negative chip results were significantly associated to better treatment outcomes in the FOLFOX-4 plus cetuximab group (*P* = 0.047).

**Conclusions:**

The results demonstrated that the WEnCA technique is a sensitive and convenient technique that produces easy-to-interpret results for detecting activated *KRAS* from the peripheral blood of cancer patients. We suggest that the WEnCA technique is also a potential tool for predicting responses in CRC patients following FOLFOX-4 plus cetuximab chemotherapy.

## Background

Ras proteins, which play a key role in cell growth, apoptosis, motility, and differentiation, are low molecular weight (21 kD) GTPases that cycle between the GDP-bound (inactive) and the GTP-bound (active) states at the plasma membrane [1,2] and bind to and activate a plethora of downstream effector proteins, including Raf kinases, phosphatidylinositol 3-kinases (PI3-K), and RalGDS family members [3-5]. The activation of mutations of the ras family is among the most common genetic events of human tumorigenesis [6]. Constitutive activations of the three canonical family members—K-ras, N-ras, and H-ras are segregated strongly by tissue type [7]. Of these, *KRAS* mutations are the most common in human tumors, including those arising from the colon and lungs [8]. In our previous research analysis of the *KRAS* mutation of lung cancer, colorectal cancer (CRC), and adrenocortical cancer, the mutation rates of these cancer tissues were found to be 37%, 26%, and 45%, respectively [9-14]. The frequency of *KRAS* mutations across a broad range of human tumors suggests the potency of the oncogenic contribution of the constitutively active form of this protein.

In recent years, due to rapid developments in targeted therapies, numerous monoclonal antibodies and molecular drugs that have been developed and applied clinically, such as Iressa and Cetuximab. Many reports show that *KRAS* mutations are highly specific negative predictors of response to epidermal growth factor receptor-tyrosine kinase inhibitors (EGFR-TKIs) monotherapy in advanced non-small-cell lung cancer (NSCLC) and similarity to anti-EGFR monoclonal antibodies alone or in combination with chemotherapy in metastatic colorectal cancer (mCRC) [15-18]. Therefore, the efficient, accurate, and fast analysis for detecting *KRAS* mutations status in cancer patients before selecting such type of targeted therapy is considered quite important.

So far, therapeutic targets such as *HER2/neu*, *EGFR*, *KRAS*, and *BRAF* are analyzed using polymerase chain reaction (PCR) combining direct sequencing, fluorescence in situ hybridization (FISH), real-time PCR, and other methods. These methods have disadvantages, such as inadequate sensitivity and the need to collect patients’ cancer tissues as a specimen, which make medicinal-effect evaluations prior to clinical treatment difficult. When the tumor size is too small, when the tumor has been removed by resection, or when the tumor has metastasized, no tumor tissues can be obtained for such analyses. In previous studies, we successfully constructed the Activating *KRAS* Detection Chip for detecting *KRAS* activation from peripheral blood, and demonstrated that there was a high level of correlation between activating *KRAS* and *KRAS* mutations [10,19]. Since the target genes on the chip were originally selected from a microarray which had been used to distinguish between adrenocortical tumor tissues with mutant *KRAS* and normal controls [19], and since the detection accuracy was validated as 93.85% in that study, the chip is reasonably referred to as *KRAS* detection chip. On the other hand, a correlation between *KRAS* mutations and poor responses to EGFR targeted treatment was also found [20,21]. For this reason, the detection of activating *KRAS* could be used to predict the response to EGFR targeted treatment.

**Figure 1:**
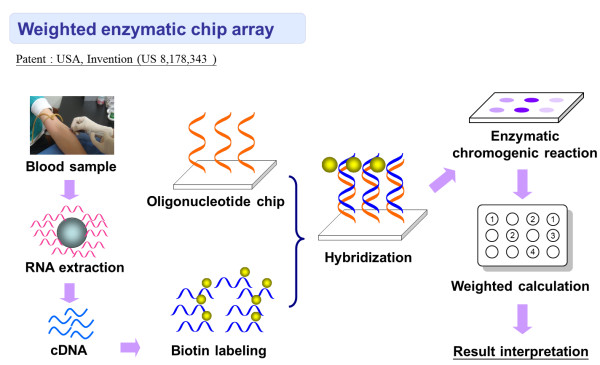
**The operating procedure of weighted enzymatic chip array (WEnCA).** The operating procedure of weighted enzymatic chip array (WEnCA) includes RNA purification by magnetic beads, cDNA labeling with Biotin, enzymatic chromogenic reaction, and weighted calculation for result interpretation of the chip.

Although this technique provides a convenient way of using peripheral blood directly for detecting *KRAS* activation and has achieved major breakthroughs in clinical applications, its sensitivity is only approximately 84% [19]. The aim of this research is to improve this technique by using a weighted enzymatic chip array (WEnCA) platform (Figure 1), in which the weighted scores are added according to the relevance of each gene to activating *KRAS* mutations. The 22 candidate genes on the Activating *KRAS* Detection Chip are given different weighted values, based on the performance after *KRAS* activation, in order to develop a detection platform that is more sensitive, accurate, and easier to read than the former technique that did not include weighted calculation which was also established by our research team [22]. The weighted calculations of genes on the Activating *KRAS* Detection Chip simplify the interpretation of results, due to the widened gap between the positive and negative results. In the current study, we collected 390 peripheral blood samples from NSCLC and CRC patients to evaluate their clinical *KRAS* activation using the WEnCA technique to analyze the sensitivity, specificity, and diagnostic accuracy of WEnCA. In advance, we analyzed the correlations among relapse status, chemotherapy (oxaliplatin, folinic acid, and fluorouracil (FOLFOX-4) chemotherapy) plus cetuximab status and chip results for 88 patients with Union for International Cancer Control (UICC) stage III CRC. The results provided evidence that using the Activating *KRAS* Detection Chip in clinical contexts has the potential to increase the accuracy of chemotherapy efficacy predictions for stage III CRC patients receiving chemotherapy plus cetuximab treatment.

## Materials and methods

### Clinical samples collection

Initially, cancerous tissues from 390 randomly selected cancer patients, including 210 NSCLC patients and 180 CRC patients, were enrolled into this study. The data of these 390 cancer patients were used to analyze the sensitivity, specificity, and diagnostic accuracy of WEnCA. Furthermore, we enrolled 88 stage III CRC patients to investigate the clinical application of chip results and their correlations with CRC relapse status in patients receiving FOLFOX-4 plus cetuximab or FOLFOX-4 alone.

Cancerous tissues and corresponding preoperative peripheral blood samples (5 ml) from 210 randomized NSCLC patients and 180 randomized CRC patients undergoing radical resection were investigated using WEnCA. All of these patients had undergone surgical resection, with NSCLC and CRC pathologies diagnosed in two hospitals, including Fooyin University Hospital and Kaohsiung Medical University Hospital. To avoid the contamination of skin cells, blood samples were taken through an intravenous catheter before surgery, and the first few milliliters of blood were discarded. Total RNA was immediately extracted from the peripheral whole blood and then served as templates for complementary DNA (cDNA) synthesis. Tissue specimens were collected immediately after surgical resection, frozen instantly in liquid nitrogen, and stored in a freezer at -80°C until analysis. Sample acquisition and use were approved by the Institutional Review Boards of the two hospitals.

Moreover, we included 88 stage III CRC patients who were treated postoperatively with adjuvant FOLFOX-4 plus cetuximab or with FOLFOX-4 chemotherapy only. The FOLFOX-4 plus cetuximab regimen consisted of biweekly cetuximab at a dose of 500 mg/m^2^ in a two-hour infusion, followed by FOLFOX-4 chemotherapy on day 1 of a 14-day cycle. The FOLFOX-4 treatment consisted of 85 mg/m^2^ of oxaliplatin concurrent with 200 mg/m^2^ of leucovorin, both as a two-hour infusion on day 1, followed by a 400 mg/m^2^ bolus of 5-FU and a continuous infusion of 600 mg/m^2^ of 5-FU over 22 -hours, was repeated every 2 weeks for 12 cycles in total. Postoperative surveillance consisted of a medical history, physical examination, and laboratory studies every 3 months. Abdominal ultrasonography or CT was performed every 3 months during chemotherapy. After the chemotherapy was completed, abdominal ultrasonography or CT was performed every 6 months. Chest radiography and a total colonoscopy were performed once a year. The enrolled patients were followed up at 3-month intervals for 2 years and at 6-month intervals thereafter. Relapse was defined as any local recurrence or distant metastases within 36 months after the adjuvant chemotherapy. Then, we compared those blood specimen chip results with the relapse status for these patients.

### DNA extraction and direct sequencing

Genomic DNA was isolated from the surgically resected primary tumor tissues using a proteinase-K (Stratagene, La Jolla, CA, USA) digestion and phenol/chloroform extraction procedure, according to the method of Sambrook [23]. To identify mutations of the KRAS genes in cancerous tissues, polymerase chain reaction (PCR) analysis was performed. The oligonucleotide primers for KRAS exons 1 and 2 were used (Table 1). Briefly, the PCR amplification of DNA samples (20 ng) was performed in a 50 ul reaction volume with a final concentration of 19 PCR buffer [10 mmol/l Tris–HCl (pH 8.3), 1.5 mmol/l MgCl2, 50 mmol/l KCl, and 0.01% gelatin], 100 mmol/l deoxynucleotide triphosphate (Promega), and 5 U (1 U/ul) BioTools DNA polymerase (Biotechnological and Medical Laboratories, S.A., Madrid, Spain) for each reaction. The PCR products were purified by a QIAEX II gel extraction kit (Qiagen Inc., Valencia, CA, USA) and then subjected to sequencing using a double-stranded cycle sequencing system (Gibco-BRL, MD, USA). The purified products were then sequenced directly with a T7 promoter/IRD800 (LI-COR, Lincoln, NE, USA), which is a T7 promoter primer (Table 1) labelled with a heptamethine cyanine dye, using DNA polymerase incorporating infrared fluorochrome (IRD)-labelled dATP for sequencing reaction. To detect and analyse the sequencing ladder, an automated DNA electrophoresis system (Model 4200; LICOR) with a laser diode emitting at 785 nm and fluorescence detection between 815 and 835 nm was used. Following the loading of samples, electrophoresis was executed at a constant voltage of 2,000 V with the gel heated to 50°C. Data collection and image analysis were performed using an IBM486 (Model 90) with the Base Image IR software supplied with the model 4200 DNA sequencer.

**Table 1:** **Nucleotide sequences of oligonucleotide primers used for PCR and sequencing of *KRAS***

		**Sequences**		**PCR product (bp)**
**Gene**	**Exon**	**Forward primer (5′ → 3′)**	**Reverse primer (5′ → 3′)**	
*KRAS*	1	*TAATACGACTCACTATAGGGAGATATGTTGAGGGCCCATCTCTC*	*TCCTAGGTCAGCGCAACCAAAT*	131
*KRAS*	2	*TAATACGACTCACTATAGGGTTCCTACAGGAAGCAAGTAG*	*CACAAAGAAAGCCCTCCCCA*	148
Sequencing primer		*CCCTATAGTGAGTCGTATTA*		

### Total RNA extraction and first strand cDNA synthesis

Total RNA was extracted from the fresh whole blood of cancer patients using the GeneCling® Enzymatic Gene Chip Detection Kit (MedicoGene Biotechnology Co., Ltd., Los Angeles, CA, USA). Purified RNA was quantified by OD 260 nm using an ND-1000 spectrophotometer (NanoDrop Technologies, Wilmington, DE, USA) and quantitated by Bioanalyzer 2100 (Agilent Technologies, USA). First-strand cDNA was synthesized from total RNA using a GeneCling® Enzymatic Gene Chip Detection Kit. Reverse transcription was performed in a reaction mixture consisting of a 3 μg/ml oligo (dT) 18-mer primer, 1 μg/ml random 6-mer primer, 100 mmol/l deoxyribonucleotide triphosphate, 200 units of Reverse Transcriptase MMLV, and 25 units of ribonuclease inhibitor. The reaction mixtures with RNA were incubated at 42°C for a minimum of 2 h, heated to 95°C for 5 min, and then stored at -80°C until analysis.

### Preparation of activating *KRAS* detection chip

The procedure of the membrane-array method for gene detection was performed based on our previous study [19]. Visual OMP3 (Oligonucleotide Modeling Platform, DNA Software, Ann Arbor, MI, USA) was used to design probes for target genes and *β-actin*. The oligonucleotide sequences of 22 target genes for Activating *KRAS* Detection Chip are listed in Table 2. The newly synthesized oligonucleotide fragments were dissolved in distilled water to a concentration of 100 mM, and applied to a BioJet Plus 3000 nL dispensing system (BioDot Inc., Irvine, CA, USA), which blotted the target oligonucleotide; the β-actin control was used sequentially (0.05 μL per spot and 1.5 mm between spots) on a SuPerCharge nylon membrane (Schleicher and Schuell, Dassel, Germany) in triplicate. After rapid drying and cross-linking procedures, the preparation of the membrane array was completed. The expression levels of each gene spot measured by the WEnCA method were quantified and then normalized based on reference gene (*β-actin*) density. We have defined as an overexpressed gene spot as a case wherein the observed normalized spot density was 2 or more.

**Table 2:** Oligonucleotide sequences of target genes

**Gene**	**Oligonucleotide sequences (5′ → 3′)**
*ATP2A2*	*ACCCGGACTTTGAAGGCTTGGATTGTGCAATCTTTGAATCCCCATACCCG*
*ATP6V0B*	*CATCGGCCATCGGAACTACCATGCAGGCTACTCCATGTTTGGGGCT*
*BCL2*	*ACAACATCGCCCTGTGGATGACTGAGTACCTGAACCGGCACCTGCACA*
*CALM2*	*GAAGCATTCCGTGTGTTTGATAAGGATGGCAATGGCTATATTAGTGCTGCAGAACTTCG*
*CEBPB*	*CCGCCTGCCTTTAAATCCATGGAAGTGGCCAACTTCTACTACGAGGCGGA*
*CLSTN1*	*TCTCTCCTCTCTCCTCCCCAGAGCACCCCCTGCCATCAGGGGGGTTGAAA*
*COL4A1*	*GCAAATGTGACTGCCATGGAGTGAAGGGACAAAAGGGTGAAAGAGGCCTC*
*CXCL11*	*GTTCAAGGCTTCCCCATGTTCAAAAGAGGACGCTGTCTTTGCATAGGCCC*
*CXCR4*	*CCCCATCCTCTATGCTTTCCTTGGAGCCAAATTTAAAACCTCTGCCCAGCAC*
*CYR61*	*CAGCAGCCTGAAAAAGGGCAAGAAATGCAGCAAGACCAAGAAATCCCCCG*
*DVL3*	*CGTCACCTTGGCGGACTTTAAGGGCGTTTTTGCAGCGACCCAGCTATAAGT*
*E2F4*	*TGAGATCACAGTGAGTGGCGGCCCTGGGACTGATAGCAAGGACAGT*
*ETS1*	*TGGAGCAGCCAGTCATCTTTCAACAGCCTGCAGCGTGTTCCCTCCTATGA*
*H2AFZ*	*CGTGGAGATGAAGAATTGGATTCTCTCATCAAGGCTACAATTGCTGGTGGTGGTGTC*
*L1CAM*	*CCTTCCTGGTGGTGTCCAACACGTCCACCTTCGTGCCCTATGAGATCAAA*
*LRP1*	*ATGCCTGTGAAAACGACCAGTATGGGAAGCCGGGTGGCTGCTCTGACAT*
*RAP1B*	*GGAAGATGAAAGAGTTGTAGGGAAGGAACAAGGTCAAAATCTAGCAAGACAATGGAACAACTGTG*
*RPL30*	*GCTCCAACTCGTTATGAAAAGTGGGAAGTACGTCCTGGGGTACAAGCAGAC*
*SLC25A5*	*TCTGATGGGATTAAGGGCCTGTACCAAGGCTTTAACGTGTCTGTGCAGGG*
*SPP1*	*GTGGACAGCCAGGACTCCATTGACTCGAACGACTCTGATGATGTAGATGAC*
*TAF12*	*CAGCACCCCTCCACAAGGCTCCATGGCCAATAGTACTGCAGTGGTAAAGA*
*TBX19*	*TCATCTGCTCAATGTGGTGGAGAGTGAGCTTCAGGCAGGGAGGGAAAAAG*
*β-actin*	*TGCATTGTTACAGGAAGTCCCTTGCCATCCTAAAAGCCACCCCACTTCTCTCTAAGGAGA*

### Preparation of Biotin-labeled cDNA targets and hybridization

**Figure 2:**
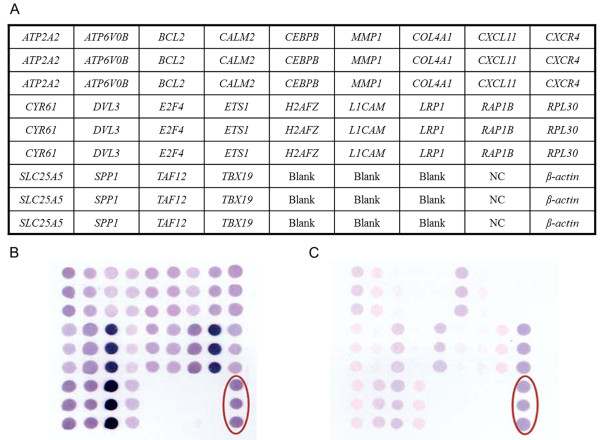
**The schematic representation of an Activating *KRAS *Detection Chip and the analytic results in the peripheral blood specimen. (A)** The schematic representation of an Activating *KRAS* Detection Chip with 22 candidate genes, one positive control (*β-actin*), one negative control (*Oryza sativa* sequence), and the blank control (dd water). Oligonucleotide fragments are blotted on membranes in triplicate. The expression levels of each gene spot were quantified and then normalized based on reference gene (*β-actin*) density which the spots are within the red circle of each image. We defined an overexpressed gene spot when the normalized spot density was 2 or more. Each overexpressed spot was then multiplied by respective weighted values ranging from 1 to 4 based on the performance after *KRAS* activation to calculate the total score of the chip. When the total score was higher than cutoff value 20, the chip result were considered to be positive. **(B)** Detectable *KRAS* oncogene from circulating RNA in the peripheral blood. **(C)** Undetectable *KRAS* oncogene from circulating RNA in the peripheral blood.

First-strand cDNA were applied for biotin labeling, and the biotin labeled probes then hybridized with the Activating *KRAS* Detection Chip. The hybridized chip followed washing, blocking and color development procedures using a GeneCling® Enzymatic Gene Chip Detection Kit. The hybridized arrays were then scanned with an Epson Perfection 1670 flatbed scanner (SEIKO EPSON Corp., Nagano-ken, Japan). Subsequent quantification analysis of the intensity of each spot was executed using AlphaEase® FC software (Alpha Innotech Corp., San Leandro, CA, USA). Spots consistently carrying a factor of two or more were considered to be differentially expressed. A deformable template extracted the gene spots and quantified their expression levels by determining the integrated intensity of each spot after background subtraction. The fold ratio for each gene was calculated as follows: spot intensity ratio = mean intensity of target gene / mean intensity of *β-actin*. Figure 2 provides the schematic representation of the membrane array with 22 candidate genes, one positive control (*β-actin*), one negative control (*Oryza sativa* sequence), and the blank control (dd water). As the concentration of *β-*actin was diluted 10–20 fold diluted for spotting, it would only present the medial-low expression level. According to the procedure, the chromogenic reaction was stopped depending on the appearance of the strongest spots; thus, the expression of *β-*actin on each chip would not be the same even under a longer chromogenic development procedure. Since *β-*actin acted as the internal control on each chip, all the other spots were then normalized based on the density of *β-actin* to reduce the individual differences.

### Chip interpretation (WEnCA method)

A deformable template extracted the gene spots and quantified their expression levels by determining the integrated intensity of each spot after background subtraction. The fold ratio of each gene was normalized based on reference gene (*β-actin*) density as follows: spot intensity ratio = mean intensity of target gene/mean intensity of *β-actin*. Normalized spot density carrying a factor of 2 or more was considered to be a differentially overexpressed gene. Each overexpressed spot was then multiplied by respective weighted values ranging from 1 to 4 based on the performance after *KRAS* activation to calculate the total score of the chip. When the total score was higher than cutoff value 20 which was determined through Receiver Operating Characteristic (ROC) Curve in our previous study, the chip was defined as positive result [22].

### Statistical and database analysis

All statistical analyses were performed using the Statistical Package for the Social Sciences version 18.0 (SPSS, Inc., Chicago, IL, USA). A chi-square test was used to analyze the association between WEnCA and direct sequencing for activated *KRAS* detection in peripheral blood and tumor tissues. The sensitivity, specificity, positive predictive value (PPV), negative predictive value (NPV) and accuracy of the WEnCA platform were evaluated. The chip results and the clinical pathological features of NSCLC and CRC patients, and the relapse status and cetuximab medication status between the two groups (positive chip result versus negative chip result) were compared using chi-square test. A p-value of less than 0.05 was considered statistically significant.

## Results

### Clinicopathological features of cancer patients

We used data from a sample consisting of 206 men (52.8%) and 184 women (47.2%). The mean age was 64 years (range 37–87 years) for the 210 NSCLC patients and 65 years (range 29–79 years) for the 180 CRC patients. The clinicopathologic characteristics of these cancer patients are listed in Table 3; 202 patients were subsequently diagnosed with stage I-II cancers, and 188 were subsequently diagnosed with stage III-IV cancers.

**Table 3:** Clinicopathologic characteristics of 210 non-small-cell lung cancer patients and 180 colorectal cancer patients

	**Lung cancer**	**Colorectal cancer**
**Variables**	**number (%)**	**number (%)**
Gender		
Male	112 (53.3)	94 (52.2)
Female	98 (46.7)	86 (47.8)
Age (years)		
<65	115 (54.8)	85 (47.2)
≥65	95 (45.2)	95 (52.8)
Stage (AICC)^a^		
I + II	109 (51.9)	93 (51.7)
III + IV	101 (48.1)	87 (48.3)
T stage		
T1 + T2	149 (70.9)	44 (24.4)
T3 + T4	61 (29.1)	136 (75.6)
Lymph node metastasis		
No	94 (44.8)	93 (51.7)
Yes	116 (55.2)	87 (48.3)

^a^American Joint Committee on Cancer.

### The association between WEnCA and direct sequencing for activated *KRAS* detection in peripheral blood and tumor tissues

To establish the capabilities of the WEnCA platform for the clinical detection of *KRAS* activation in blood samples, we collected 390 samples of peripheral blood from pathology-proven NSCLC and CRC patients. All specimens were tested with the Activating *KRAS* Detection Chip using the WEnCA method (Table 4). The paired cancer tissue of 390 samples then served to detect *KRAS* mutational status by direct sequencing. There were 127 cancer tissues with *KRAS* mutants. The mutation sites are distributed in codon 12, 13, 15, 18, 31, and 60. Among them, 117 were positive through WEnCA. Moreover, among the 263 paired cancer tissues with wild type *KRAS*, 249 were negative through WEnCA. After statistical analysis, the sensitivity, specificity, PPV, NPV and accuracy of WEnCA were 92.13%, 94.68%, 89.31%, 96.14% and 93.85%, respectively (p <0.001).

**Table 4:** **The sensitivity, specificity, and accuracy of weighted enzymatic chip array for *KRAS *** **mutation detection**

	**Direct sequencing**^**a**^								
	**Mutation (N = 127)**	**Wild type (N = 263 )**	**Total**	**Sensitivity**	**Specificity**	**PPV**^**c**^	**NPV**^**d**^	**Accuracy**	**P-value**
				**(95% CI)**	**(95% CI)**	**(95% CI)**	**(95% CI)**	**(95% CI)**	
WEnCA^b^									
Positive	117	14	131	92.13%	94.68%	89.31%	96.14%	93.85%	<0.001
Negative	10	249	259	(86.11-95.67%)	(91.26-96.8%)	(82.86-93.53%)	(93.04-97.89%)	(91.47-96.23%)	

^a^*KRAS* in tumor tissues (direct sequencing).

^b^*KRAS* in peripheral blood (weighted enzymatic chip array; WEnCA).

^c^PPV: positive predictive value.

^d^NPV: negative predictive value.

95% CI: 95% confidence interval.

### The association between the calculation results of WEnCA and the clinicopathological features

The mean positive gene number and the mean total score of the positive chip by WEnCA associated significantly with the AJCC stage, T stage, and distant metastasis (p <0.05) (Table 5).

**Table 5:** The association between the mean positive gene number and the mean positive total score by WEnCA and the clinicopathological features of cancer patients

		**WEnCA**		
		**Positive number**	**Mean positive total score**	**P value**
**Variables**	**Total**	**117**	**46.86**	
Stage (AJCC)^a^				
I + II	202	41	41.62	< 0.0001
III + IV	188	76	48.73	
T stage				
T1 + T2	193	75	43.93	< 0.0001
T3 + T4	197	42	50.37	
Lymph node metastasis				
No	187	50	47.67	0.177
Yes	203	67	46.64	
Distant metastasis				
No	297	79	41.21	0.009
Yes	93	38	54.72	

^a^ American Joint Committee on Cancer.

### The association between clinical relapse status and WEnCA results in stage III CRC patients treated with or without cetuximab

A significant association was found between the chip results, cetuximab treatment and clinical outcome that 51 patients with negative chip results received cetuximab treatment, and 34 (66.7%) of those patients had no relapse (p = 0.047). On the other hand, 6 (75%) of the 8 patients with positive chip results who chose to receive cetuximab treatment, most had a relapse (6 patients, 75%) (Table 6). Otherwise, no significant relationship was found in patients who were treated with FOLFOX-4 alone (p = 0.633). Half of 6 patients with positive chip results who received FOLFOX-4 alone had a relapse. Similarly, 16 (48.5%) patients with positive chip results received FOLFOX-4 alone, and half of them had a relapse (Table 6).

**Table 6:** **The association between chemotherapy regimen**, **relapse status**, **and WEnCA result in 88 stage III CRC patients**

**Chemotherapy regimen (N)**	**WEnCA (N)**	**Relapse**	**No relapse**	***P-value***
		**N = 42 (%)**	**N = 46 (%)**	
FOLFOX-4 plus Cetuximab (N = 59)	Positive (N = 8)	6 (75)	2 (25)	0.047
	Negative (N = 51)	17 (33.3)	34 (66.7)	
FOLFOX-4 (N = 29)	Positive (N = 6)	3 (50)	3 (50)	0.633
	Negative (N = 23)	16 (48.5)	7 (21.2)	

## Discussion

Previous studies [24,25] showed that the benefits of the anti-EGFR mAb cetuximab among patients with metastatic colorectal cancer are limited to those patients who have colorectal tumor tissues with wild-type *KRAS* genes, and *KRAS* genes with mutations are essentially insensitive to EGFR inhibitors. In particular, *KRAS* genotyping of primary tumor tissues or metastatic lesions is strongly recommended by the National Comprehensive Cancer Network (NCCN) Clinical Practice Guidelines in Oncology version 3 (2008) in patients with mCRC prior to any therapy that includes anti-EGFR mAbs [26]. Therefore, it is important to identify mCRC patients who harbor *KRAS* mutants prior to the addition of such expensive targeted therapies to standard chemotherapy.

*KRAS* genotyping highlights the value of banking tumor specimens obtained from primary tumors or a metastasis. In most of these studies, *KRAS* genotyping was performed on primary colorectal cancers, whereas anti-EGFR antibodies were used to treat the metastatic disease. This strategy might, at least in certain circumstances, present two limitations [27]. First, systematic *KRAS* genotyping in metastatic colorectal cancer patients might be hampered in the future, at least for some patients, by the difficulty of obtaining tumor samples suitable for molecular analyses. Second, considering the genetic heterogeneity of colorectal cancers [28,29], the absence of detectable *KRAS* mutations in the primary tumor may not formally exclude the presence of a *KRAS* mutation in metastases, and consequently, additional tumor samples need to be examined in order for *KRAS* mutations to correctly predict the *KRAS* status in metastatic lesions. Hence, an alternative method for detecting *KRAS* gene mutations in these metastatic colorectal cancer patients treated with anti-EGFR is needed. We used this chip to detect the activating *KRAS*, not to directly identify its mutation status. There are many mutation sites on the *KRAS* gene, including codons 12, 13, 15, 18, and 31, among others, but while some mutations activate *KRAS*, others do not. In our earlier reports [10,19], we found that blood samples with *KRAS* mutations in codon 31 always showed negative results in this chip assay. The possible reason for this finding is that the mutation site of these codons cannot activate *KRAS*, which may explain the discordance in KRAS status between tumor and blood samples.

A previous study reported that *KRAS* mutations in a tumor may not be detected in the bloodstream and suggested that this non-detection may be caused by the low DNA concentration, as well as the heterogeneity [30]. Since the principle of the Activating *KRAS* Detection Chip is to detect the expression of multiple downstream genes from *KRAS*, it could reveal the integral situation of activating *KRAS* instead of detecting the status from a single marker, which may be undetectable because of detection limitations, and in this way also overcome the heterogeneity issue.

Our recently developed membrane-array-based multi-marker assay can detect activating *KRAS* mutations in the circulating RNA in the peripheral blood of patients with various malignancies, including colorectal cancer, achieving considerable sensitivity, specificity, and accuracy when compared to the direct sequencing of tumor tissues [19]. The results of the current study demonstrate that WEnCA is a sensitive and convenient technique for detecting activated *KRAS* from the peripheral blood of NSCLC and CRC cancer patients. In fact, the sensitivity of WEnCA reached 92.13% and the specificity reached 94.68% in this study, and the similar results which the sensitivity, specificity, and accuracy were all above 92% in previous studies using WEnCA platform [31,32].

Although the Next Generation Sequencing (NGS) is a rapidly developed high-throughput technique that improves the sensitivity and reduces the cost of Sanger sequencing, the difficulty in tumor tissue specimen collection still cause the limitation for this method [33]. Even NGS can be applied to RNA sequencing using blood sample [34], the data analysis and interpretation is quiet complicated especially compared with WEnCA platform which is easy to interpret and only needs basic calculation. Moreover, the overall cost of NGS is higher than membrane-based WEnCA platform currently.

The identification of activated *KRAS* status could be extremely useful in selecting feasible CRC patients for cetuximab therapy, allowing some patients to avoid unnecessary treatment. In the present study, the relapse rate was only 17/51 (33.3%) in stage III CRC patients with negative chip results who received cetuximab therapy; on the other hand, the rate was 75% among patients with positive chip results. There were prominent associations between the chip results and relapse status, and these associations could therefore be used as a pre-cetuximab therapy predictor for clinical outcomes of stage III CRC. This finding could be useful in the future for identifying individual risk and developing alternative therapeutic strategies.

## Conclusions

This present study indicated that a panel of molecular markers could be applied, in conjunction with our constructed membrane-array method with weighted calculation, to detect activating *KRAS* status from circulating RNA in the peripheral blood of NSCLC and CRC patients. The Activating *KRAS* Detection Chip using WEnCA technique could also be a potential aid in clinical predictions for obtaining better cetuximab response prediction models. The results of the present study suggest that such technique could be used to distinguish between CRC patients who will respond to cetuximab treatment and those who will not. That being said, further studies involving larger sample sizes and even multiple centers are needed to verify these results.

## Consent

Written informed consent was obtained from the patient for the publication of this report and any accompanying images.

## Competing interests

The authors declare that they have no competing interests.

## Authors' contributions

MYH analyzed the data and drafted the manuscript. MYH, HCL, LCY, JYC, JJH and CPH made contributions in data acquisition, molecular genetic analyses, statistical analyses and data interpretation. MYH, JYW and SRL participated in the design and coordination of the study. All authors read and approved the final manuscript.
